# Histopathological analysis of the skin of renal transplant recipients submitted to three different immunosuppression regimens^☆^

**DOI:** 10.1016/j.abd.2024.07.016

**Published:** 2025-04-07

**Authors:** Maria Victória Quaresma, Luiz Sergio Azevedo, Elias David-Neto, Mírian Nacagami Sotto

**Affiliations:** a Division of Dermatology, Hospital das Clínicas, Faculty of Medicine, Universidade de São Paulo, São Paulo, SP, Brazil; b Renal Transplant Service, Clinical Hospital, Faculty of Medicine, Universidade de São Paulo, São Paulo, SP, Brazil; c Department of Pathology, Faculty of Medicine, Universidade de São Paulo, São Paulo, SP, Brazil

**Keywords:** Histopathology, Immunosuppression therapy, Kidney transplantation, Skin neoplasms

## Abstract

**Background:**

Renal transplant recipients (RTRs) use a combination of immunosuppressive agents: a corticosteroid; a calcineurin inhibitor (cyclosporine or tacrolimus) and an antimetabolic agent (azathioprine [AZA] or a mycophenolic acid precursor [MPA] ‒ Mycophenolate mofetil or sodium) or an mTOR inhibitor (mTORi) ‒ sirolimus or everolimus. These treatments increase the incidence of various neoplasms, especially non-melanoma skin cancers (NMSCs).

**Objectives:**

To evaluate the histopathological alterations in the skin of the RTRs under three different immunosuppressive regimens: one mTORi (sirolimus or everolimus); or one antimetabolic agent (MPA or AZA), comparing them by groups and with healthy controls.

**Methods:**

This was a cross-sectional comparative study of 30 patients selected from the Renal Transplant Service and divided into three groups: mTORi (n = 10), MPA (n = 10), and AZA (n = 10). The control group consisted of 10 immunocompetent non-transplanted volunteers. All RTRs were using tacrolimus and prednisone. Each participant underwent two biopsies of intact skin: one in a sun-protected and another in a sun-exposed area. The specimens were analyzed without previous information on which group they belonged to.

**Results:**

The most significant histopathological change was thinning of the epidermis in the mTORi group, both in photoexposed and photoprotected skin.

**Study limitations:**

The study was conducted on a limited number of patients, which may influence the representativeness of the results.

**Conclusions:**

Only RTRs treated with mTORi presented interruption of epidermal proliferation. These findings help to understand the influence of these different types of immunosuppressive regimens and their subsequent potential effects on carcinogenesis.

## Introduction

Organ transplantation, particularly in renal transplant recipients (RTRs), has made extraordinary progress largely due to the optimization of immunosuppressive regimens, significantly contributing to reducing graft rejection rates and increasing survival, both for the organ and the patient. The strategies used to reduce immunogenicity involve the use of drugs that block the action of immune system cells.^1^ In the maintenance phase, a combination of three therapeutic classes is used: a corticosteroid (prednisone) combined with a calcineurin inhibitor (CNI; tacrolimus [TAC] or cyclosporine [CsA]) and an antiproliferative agent: azathioprine (AZA) or a mycophenolic acid precursor (MPA) (mycophenolate mofetil [MMF] or mycophenolate sodium [MPS]). Alternatively, one of these agents can be replaced by a mammalian target of rapamycin inhibitors (mTORi), such as everolimus (EVE) or sirolimus (SRL, also called rapamycin). Subsequent changes, such as minimization and conversion, are only recommended when due to adverse events or related to efficacy or safety failures.^2^

The use of immunosuppressive drugs has several side effects, including cutaneous ones, particularly non-melanoma skin cancers (NMSCs), responsible for approximately 90% of all skin cancers in RTRs, which have different epidemiology and pathogenesis compared to the general population.^3^, ^4^, ^5^, ^6^, ^7^, ^8^, ^9^ With a much higher incidence in RTRs, NMSCs generate substantial morbidity and mortality due to their common recurrence and higher metastatic potential. Among NMSCs, squamous cell carcinoma (SCC) is the most commonly found type, surpassing basal cell carcinoma (BCC).^10^ The SCC/BCC ratio is 3-4:1, with the inverse being observed in the general population. The incidence rates of NMSCs increase steadily with time after transplantation. It is estimated at about 2.25% in one year to 4.95% in two years, 7.43% in three years, and then increases to 10%-27% and 40%-60% after 10 and 20 years of immunosuppression, respectively.^11^ In large series, it is observed that RTRs show an estimated 65 to 250-fold increase in the incidence of SCC and a 10-fold increase in the incidence of BCC when compared to immunocompetent populations.^12^, ^13^, ^14^ This epidemiological reversal increases with greater sun exposure and post-transplant time lapse.^15^, ^16^, ^17^, ^18^, ^19^ RTRs have an increased risk of developing skin cancer in photoexposed skin areas, as well as immunocompetent individuals.

Different mechanisms by which drugs may contribute to the development of skin cancer include systemic immunosurveillance impairment and a direct oncogenic effect.^14^, ^16^, ^20^ An aggravating factor is exposure to ultraviolet radiation (UVR), because, in addition to interacting with certain drugs by increasing skin photosensitivity, it causes gene mutations and exerts local or systemic immunosuppression.^21^, ^22^, ^23^ Most tumors occur on photoexposed skin, in fair-skinned individuals, and in those with a history of chronic sun exposure and/or episodes of sunburn in childhood.^24^, ^25^, ^26^ UVR causes genetic mutations in epidermal keratinocytes, which affect cell cycle regulation, suppress the immune response, inhibit the expression and activity of antigen-presenting cells, and compromises the antigen recognition of neoplastic cells in RTRs. The geographic location where transplant recipients reside is an aggravating factor, due to the degree of sun exposure. Patients living in countries with high sun exposure, such as Australia, have skin cancer risks of 45% and 70%, after kidney transplantation at 11 and 20 years, respectively, and those living in countries with limited sun exposure, such as the Netherlands, have post-transplant risks of 10% and 40% at 10 and 20 years, respectively.^13^, ^27^, ^28^ Other factors that put the general population at risk for NMSCs are also associated with higher risk in RTRs. These include older age, mainly due to the cumulative rate of sun exposure; fair skin (low phototypes, Fitzpatrick I–III), history of prior skin cancer, and actinic keratoses. Additional risks include the immunosuppressive treatment duration, immunosuppression intensity, older age at the time of transplantation, depletion of CD4 cells in the blood, and human papillomavirus infection.^5^, ^14^, ^29^, ^30^, ^31^, ^32^

Before the discovery of the skin immune defenses, the cutaneous interface was seen only as a passive barrier between the individual and the environment. In recent decades it has become evident that the mechanical aspects of epidermal defense are reinforced by a versatile and robust immune surveillance system.^33^ The specific role of immunosuppressive drugs in the development of SCCs has been the subject of many studies in recent years. It is presumed that immunosurveillance impairment contributes greatly to the higher incidence of these neoplasms. The pro-oncogenic effects of AZA have been known since the early years of organ transplantation. The oncogenic effects of MPA precursors are not clearly defined.^34^, ^35^

The action mechanisms of the three immunosuppressants assessed in this study are summarized below.^36^

AZA is a purine analog that is incorporated into cellular DNA, where it inhibits purine nucleotide synthesis and interferes with RNA synthesis and metabolism. AZA leads to the accumulation of its metabolite 6-thioguanine nucleotide in the DNA chain, transforming it into a chromophore that absorbs light in the UVR-A spectrum and thus is able to function as a source of oxidative photoproducts. A mechanism of indirect damage can also be identified through the inhibition of repair mechanisms in the keratinocytes of the epidermis and the consequent persistence of photoproducts from UVR type B (UVR-B).

MPA is a reversible, selective, non-competitive inhibitor of inosine monophosphate dehydrogenase (IMPDH), an enzyme important in the *de novo* synthesis of purine, which acts as a catalyst in the production of *de novo* guanosine triphosphate required for lymphocyte proliferation. Blocking IMPDH inhibits many lymphocyte functions, without significantly affecting other cells. MMF is the semisynthetic 2-morpholinoethyl ester of MPA that has shown bioavailability, tolerability, and efficacy. Adverse effects on the gastrointestinal tract are frequent and to minimize them, a MPS formulation was developed, which has a gastro-resistant coating, dissolving only in the intestine. The use of these drugs is associated with a significantly lower risk of developing malignancy compared with non-MPA-based immunosuppression regimens.

mTOR is a key kinase in the process of cell division. SRL and EVE have a similar action. They bind to a plasma immunophyllin, FKBP12 (FK binding protein), forming the rapa/FKBP12 complex that inhibits mTOR (hence they are called mTOR inhibitors: mTORi). This reduces the transduction of activation signals and proliferation of lymphocyte membrane receptors, especially those associated with the interleukin-2 receptor and the CD28 lymphocyte co-stimulation receptor.

The aim of this study was to comparatively analyze the histopathological alterations in the photoprotected and photoexposed skin of renal transplant recipients using three different immunosuppressive drug regimens (mTORi, MPA, and AZA), comparing them with non-transplanted immunocompetent individuals. At the same time, an analysis was performed on the same material on the immunohistochemical expression of B lymphocytes, total T lymphocytes, T-helper lymphocytes, cytotoxic T lymphocytes and Langerhans cells, which has already been published.^37^

## Material and methods

This research was approved by the Ethics Committee for the Analysis of Research Projects of the Institution, under number 1.685.977, and is in accordance with the ethical standards of the national and international committees on experimentation on human beings (Declaration of Helsinki). Participants were selected from the population of kidney transplant recipients who were undergoing regular outpatient follow-up at the Renal Transplant Service (RTS) of the institution. Written informed consent was obtained from all subjects who agreed to participate in this study.

### Inclusion and exclusion criteria

Renal transplant patients, of both genders, aged 18 years or older, who were undergoing stable immunosuppressive regimens containing mTORi or MPA or AZA, for a minimum of 12 months and a maximum of 72 months were included. All patients were also receiving tacrolimus and prednisone. Induction therapy was carried out with thymoglobulin (ATG) or basiliximab. Renal failure influences immunological factors and may act as a potential bias on the studied outcome. For this reason, the selected patients had stable renal function, with an estimated glomerular filtration rate (eGFR) equal to or greater than 45 mL/min/1.73/73 m^2^, corresponding to stage ≤ G3a of the KDIGO (Kidney Disease: Improving Global Outcomes) classification and estimated by the CKD-EPI (Chronic Kidney Disease Epidemiology Collaboration) formula. The patients had phototypes II, III, IV or V, according to Fitzpatrick classification (1988). The patients were separated in three groups: group 1 or mTORi group: patients using mTOR inhibitors (EVE or SRL); group 2 or MPA group: using mycophenolic acid precursors (MMF or MPS); group 3 or AZA group: on azathioprine. Transplant recipients of any organ other than the kidney; skin phototypes I and VI, according to Fitzpatrick's classification (extreme phototypes could act as bias); those with previous and/or current neoplastic events; immunodeficiencies unrelated to kidney transplantation; and patients using immunosuppressants other than those indicated in this study were excluded. For comparison, healthy non-transplanted individuals (Control Group – CG) consisting of volunteers aged 18 years or older, of both genders and who had no history of skin disease, were included. Similarly, individuals with skin phototypes I and VI according to the Fitzpatrick classification, with a history of previous and/or current neoplastic events, and primary or secondary immunodeficiencies were excluded.

The following variables were investigated in all study individuals: gender, age (completed years), skin phototype, serum creatinine levels (in mg/dL) in the last three months, and corresponding eGFR. For transplant recipients, the following clinical variables were evaluated: type of induction therapy (ATG or basiliximab); type of maintenance immunosuppressant (EVE, SRL, MPS, MMF or AZA), and the duration of maintenance immunosuppression (in full months).

Two biopsies of intact skin were performed, both in patients and controls, one on the inner surface of the arm (area not exposed to solar radiation - photoprotected) and another on the dorsum of the ipsilateral hand (area exposed to sunlight - photoexposed). A 4-mm punch was used after infiltrative local anesthesia with 2% lidocaine, without vasoconstrictor. The samples were placed in 10% formaldehyde fixing solution buffered with phosphate salts at 7.4 pH. The specimens were processed by routine histological techniques and embedded in paraffin. Sequential histological sections of 4 μm thickness were made, mounted on glass slides and stained with hematoxylin-eosin.

The histopathological analysis was performed without prior knowledge of the group to which the specimens belonged. The evaluated variables were: (A) stratum corneum of the epidermis, classifying it according to the morphological type into three categories: basket weave, lamellar and compact. (B) Stratum granulosum of the epidermis, defined by the morphological aspect of the cutaneous sites of non-glabrous skin according to thickness in three categories: normal or usual thickness (presence of one to three cell layers), hypergranulosis, when there was thickening of this stratum (number of layers greater than three), and agranulosis, when this layer was absent. (C) The number of stratum spinosum cell layers in the epithelial cones (three cones/biopsy) and in the segments of the epidermis between epithelial cones (three segments/biopsy), with arithmetic means/biopsy being recorded. (D) Degree of solar elastosis, defined as the intensity of the pale or basophilic elastotic material present in the dermis as: mild (fibrillar); moderate (fibrillar and amorphous); intense (amorphous); absent (without the presence of this material in the dermis). (E) Lymphocytic inflammatory infiltrate, was categorized by the amount of cells present around superficial and/or deep vascular structures in the dermis as: mild; moderate; intense; or absent.

### Sample size calculation and statistical analysis

To determine the number of participating individuals (n), the sample size was calculated, using a significance level of 5% and a power of 80%. The calculated sample consisted of ten individuals in each group.^37^ Thus, 30 renal transplant patients and ten healthy volunteers were included. Absolute (n) and relative (%) frequencies were used for categorical variables, respectively. In these analyses, Fisher's exact test was used to compare the qualitative variables between the groups; Dunn's test with Bonferroni post-estimation for quantitative variables without normal distribution and ANOVA with Bonferroni correction for those with normal distribution. Means and standard deviations (SD) and medians and interquartile range (IQR) were used for quantitative variables. The program used was Stata*®* (StataCorp, LC) version 11.0.

## Results

### Demographic, clinical, and laboratory data

These data are summarized in Table 1.

**Table 1:** Demographic, clinical, and laboratory data.

Variables	Control	mTORi	MPA	AZA	p
**Sample size (n)**	10	10	10	10	–
**Age (mean, SD)**	44.7 (14.6)	55.5 (19.2)	49.5 (11.9)	49.6 (17.07)	0.517^a^
**Gender n (%)**					0.971^b^
Female	5 (50.0)	4 (40.0)	5 (50.0)	6 (60.0)	
Male	5 (50.0)	6 (60.0)	5 (50.0)	4 (40.0)	
**Phototype, n (%)**					
II	2 (20.0)	3 (30.0)	2 (20.0)	2 (20.0)	0.999^b^
III	3 (30.0)	3 (30.0)	3 (30.0)	3 (30.0)	
IV	3 (30.0)	2 (20.0)	2 (20.0)	2 (20.0)	
V	2 (20.0)	2 (20.0)	3 (30.0)	3 (30.0)	
**Immunosuppression time, mean (SD)**	–	42.7 (13.26)	47.7 (9.34)	42.1 (14.18)	0.550^a^
**Induction, n (%)**	–				0.893^b^
ATG	–	3 (30.0)	5 (50.0)	4 (40.0)	
Basiliximab	–	7 (70.0)	5 (50.0)	6 (60.0)	
**Creatinine mean (SD)**^d^	0.86 (0.18)	1.39 (0.26)	1.06 (0.26)	1.25 (0.32)	<0.001^a^
**eGFR, median (p.25; p.75)**	106.8 (70.4; 111.65)	48.6 (46.98; 53.35)	71.9 (60.74; 81.87)	50.4 (57.29; 91.36)	<0.001^c^

The data are presented as n, absolute number with percentages (%), mean (standard deviation) and median (p.25; p.75: 25^th^ and 75^th^ percentiles, respectively). ATG, Antithymocyte Globulin; AZA, Azathioprine Group; SD, standard deviation; mTORi, mTOR Inhibitors Group; MPA, Mycophenolic Acid Group; n, Observed absolute frequency; p, Level of statistical significance; Conventional sign used: – No numerical data is applicable; eGFR, Estimated Glomerular Filtration Rate.

a ANOVA.

b Fisher's test.

c Dunn's test with Bonferroni correction.

d Only the mTORi, MPA, and AZA groups were included in the statistical analysis, since the individuals in the control group had both functionally normal native kidneys and were excluded from the analysis.

### Histopathological evaluation results

The results of the histopathological evaluation of photoprotected and photoexposed skin sections are depicted in Table 2, Table 3.

**Table 2:** Distribution of histopathological data on photoprotected skin, according to groups.

Variables	TOTAL	Control	mTORi	MPA	AZA	p
**Number of layers**						
**Between cones** (mean, 95% CI)	5.3 (5.02; 5.58)	5.10 (4.94; 5.26)	4.40 (4.15; 4.65)	6.30 (5.72; 6.88)	5.40 (4.98; 5.82)	<0.001^a^
**In the cones** (mean, 95% CI)	7.64 (7.22; 8.07)	7.37 (6.82; 7.91)	5.97 (5.55; 6.38)	8.97 (8.39; 9.54)	8.27 (7.75; 8.78)	<0.001^a^
**Stratum corneum n (%)**						
Compact pattern	–	–	–	–	–	>0.999^b^
Basket weave pattern	31 (77.5)	8 (80.0)	8 (80.0)	8 (80.0)	7 (70.0)	
Lamellar pattern	9 (22.5)	2 (20.0)	2 (20.0)	2 (20.0)	3 (30.0)	
**Granular layer n (%)**						
Agranulosis	–	–	–	–	–	–
Usual thickness	40 (100.0)	10 (100.0)	10 (100.0)	10 (100.0)	10 (100.0)	
Hypergranulosis	–	–	–	–	–	
**Elastosis n (%)**						
Absent	13 (32.5)	4 (40.0)	3 (30.0)	3 (30.0)	3 (30.0)	>0.999^b^
Mild	27 (67.5)	6 (60.0)	7 (70.0)	7 (70.0)	7 (70.0)	
Moderate	–	–	–	–	–	
Intense	–	–	–	–	–	
**Perivascular lymphocytic inflammatory infiltrate n (%)**						
Absent	14 (35.0)	3 (30.0)	3 (30.0)	4 (40.0)	4 (40.0)	>0.999^b^
Mild	25 (62.5)	6 (60.0)	7 (70.0)	6 (60.0)	6 (60.0)	
Moderate	1 (2.5)	1 (10.0)	–	–	–	
Intense	–	–	–	–	–	

The data are presented as n, absolute number with percentages (%) and means. AZA, Azathioprine Group; SD, standard deviation; 95% CI, 95% Confidence Interval; mTORi, mTOR Inhibitors Group; MPA, Mycophenolic Acid Group; n, Observed absolute frequency; p, Level of statistical significance; Conventional sign used: – No numerical data is applicable.

a ANOVA.

b Fisher's test.

**Table 3:** Distribution of histopathological data on photoexposed skin, according to groups.

Variables	TOTAL	Control	mTORi	MPA	AZA	p
**Number of layers**						
**Between cones** (mean, 95% CI)	7.27 (6.81; 7.74)	7.23 (6.64; 7.83)	5.39 (4.87; 5.92)	8.66 (8.10; 9.23)	7.79 (7.10; 8.50)	<0.001^a^
**In the cones** (mean, 95% CI)	10.02 (9.37; 10.66)	9.63 (8.30; 10.97)	7.83 (6.92; 8.74)	11.40 (10.97; 11.83)	11.20 (9.92; 12.48)	<0.001^a^
**Stratum corneum n (%)**						
Compact pattern	30 (75.0)	8 (80.0)	6 (60.0)	9 (90.0)	7 (70.0)	0.720^b^
Basket weave pattern	3 (7.5)	–	2 (20.0)	–	1 (10.0)	
Lamellar pattern	7 (17.5)	2 (20.0)	2 (20.0)	1 (10.0)	2 (20.0)	
**Granular layer n (%)**						
Agranulosis	–	–	–	–	–	0.105^b^
Usual thickness	27 (67.5)	8 (80.0)	9 (90.0)	4 (40.0)	6 (60.0)	
Hypergranulosis	13 (32.5)	2 (20.0)	1 (10.0)	6 (60.0)	4 (40.0)	
**Elastosis n (%)**						
Absent	–	–	–	–	–	0.963^b^
Mild	–	–	–	–	–	
Moderate	12 (30.0)	3 (30.0)	3 (30.0)	4 (40.0)	2 (20.0)	
Intense	28 (70.0)	7 (70.0)	7 (70.0)	6 (60.0)	8 (80.0)	
**Perivascular lymphocytic inflammatory infiltrate n (%)**						
Absent	25 (62.5)	5 (50.0)	6 (60.0)	6 (60.0)	8 (80.0)	0.662^b^
Mild	15 (37.5)	5 (50.0)	4 (40.0)	4 (40.0)	2 (20.0)	
Moderate	–	–	–	–	–	
Intense	–	–	–	–	–	

The data are presented as mean and respective 95% Confidence Intervals. AZA, Azathioprine Group; SD, standard deviation; 95% CI, 95% Confidence Interval; mTORi, mTOR Inhibitors Group; MPA, Mycophenolic Acid Group; n, Observed absolute frequency; p, Level of statistical significance; Conventional sign used: – No numerical data is applicable.

a ANOVA.

b Fisher's test.

**Figure 1:**
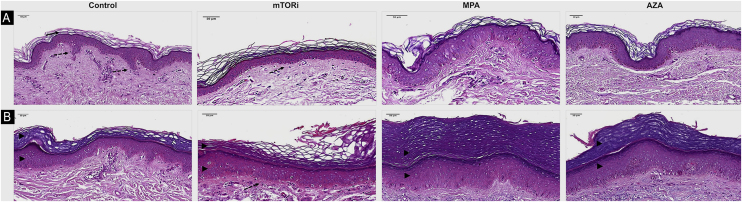
Histopathological differences related to the epidermal analysis of photoprotected (A) and photoexposed (B) skin of the control groups and renal transplant recipients submitted to immunosuppression regimens with mTOR inhibitors (mTORi), Mycophenolic Acid (MPA) and Azathioprine (AZA). The control group exhibits a basket weave pattern in the stratum corneum, typical of healthy non-acral photoprotected skin (arrow) and well-demarcated epithelial cones (discontinuous arrow). The mTORi group reveals a lamellar pattern in the stratum corneum, reduced number of epidermal cell layers, and rectification of the epithelial cones, both in photoprotected skin (discontinuous arrow; A) and in photoexposed skin (B). As a rule, photoexposed skin samples (B) exhibit thicker epidermis with hyperkeratosis and increased number of cell layers in the epithelial cones and in the epidermal segments between the cones, aspects which are more pronounced in the MPA (arrowheads) and AZA groups. (Hematoxylin & eosin, ×40; scale bar 50 m).

The photoprotected skin showed stratum corneum in "basket weave" (31/40; 77.5%) and lamellar (9/40; 22.5%) patterns. These findings were the same in the photoprotected skin of the four groups. On the other hand, the analysis of photoexposed skin fragments showed a thick and compact stratum corneum in 30 cases (30/40, 75%; Fig. 1).

The stratum granulosum of the epidermis was present with its usual thickness in the photoprotected skin of individuals in all groups (40/40; 100%; Table 2). On the other hand, the skin exposed to sunlight showed hyperplasia of the stratum granulosum of the epidermis (hypergranulosis) in 13 individuals, which corresponds to 32.5% of the individuals in this sample (13/40), six of them in the MPA group (6/10; 60%), four in the AZA group (4/10; 40%), two in the control group (2/10; 20%) and one in the mTORi group (1/10; 10%; Table 3).

**Figure 2:**
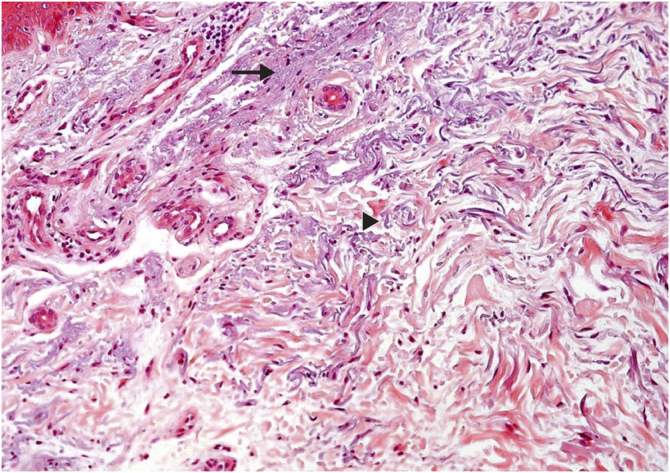
Histopathological characterization of dermal findings in photoexposed skin of a renal transplant recipient of the mTORi group. Solar elastosis is characterized by amorphous (arrow) and fibrillar (arrowhead) basophilic areas in the dermal connective tissue, where elastic fibers lose their usual characteristics. (Hematoxylin & eosin, ×200). mTORi, mTOR Inhibitors Group.

Regarding dermal alterations, the most significant finding was solar elastosis of the photoexposed skin (Fig. 2). All skin specimens from photoexposed areas showed extensive solar elastosis in the superficial dermis. Intense elastotic alteration was observed in the photoexposed skin with the presence of abundant amorphous basophilic material in the superficial dermis of 28 individuals (28/40; 70%) and moderate elastotic alteration in 12 individuals, characterized by the presence of fibrillar basophilic material and some amorphous areas in the superficial dermis (12/40; 30%; Table 3). On the other hand, in photoprotected skin, mild elastotic alteration was observed with minimal fibrillar basophilia in the papillary dermis in 27 individuals (27/40; 67.5%) and absence of dermal elastosis in 13 (13/40; 32.5; Table 2).

Regarding the inflammatory dermal infiltrate, the presence of perivascular lymphocytes was discrete, or even absent, both in photoexposed and photoprotected skin of transplant recipients and controls. The photoprotected skin revealed a lymphocytic infiltrate around the superficial dermal vessels of moderate intensity in one individual (1/40; 2.5%) of the control group and mild in 25 individuals (25/40; 62.5%). Of the latter, seven were in the mTORi group (7/10; 70%), six in the MPA group (6/10; 60%), six in the AZA group (6/10; 60%) and six in the control group (6/10; 60%; Table 2). On the other hand, in the skin exposed to sunlight, mild dermal superficial perivascular lymphocytic infiltrate was observed in 15 individuals (15/40; 37.5%). Of these, five were in the control group (5/10; 50%), four in the mTORi group (4/10; 40%), four in the MPA group (4/10; 40%), and two in the AZA group (2/10; 20%; Table 3).

There were differences in the mean number of cell layers in the epidermal cones (p < 0.001) and in the segments of the epidermis between the cones (p < 0.001) of photoprotected and sun-exposed skin among the four groups. It was observed that the highest mean values of the number of cell layers in the cones and in the epidermal segments between the cones were found in patients in the MPA and AZA groups. These findings were observed both in photoexposed skin and photoprotected skin of individuals in the MPA group.

## Discussion

Regarding demographic data, the study groups were homogeneous, with no significant differences in gender, age, or skin phototype. From the point of view of clinical data, the characteristics related to the type of induction and duration of the immunosuppressive regimen were similar in patients of the three groups. No differences were found regarding the time of exposure to the three drugs. These data thus allowed a fair comparison between the groups. The comparative analysis of the renal function in the groups (performed only in the RTRs, since there is no comparison with healthy controls who have both native kidneys) indicated that the mean serum creatinine level was higher in the mTORi group than in the AZA and MPA groups, while the eGFR was higher in the MPA group than in the mTORi and AZA groups; however, it was not possible to provide a reliable explanation for these findings.

Some morphological findings were seen equally in the photoprotected skin of the four groups, revealing mild skin changes that do not seem to be linked to photoexposure or immunosuppressive therapy. On the other hand, epidermal thinning associated with mTORi therapy was the most remarkable histopathological alteration in this study, characterized by a decrease in the epidermal thickness due to the reduction in the number of stratum spinosum layers. This was seen in both the photoprotected and photoexposed skin of this group when compared to the epidermal thickness of the other groups. This reduction in epidermal proliferation may represent a lower risk of occurrence of carcinogenic mutations, configuring epidermal thinning in the mTORi group as a potential protective effect against carcinogenesis in RTRs. This finding of epidermal thinning of the skin in the mTORi group was corroborated by a study that showed a reduction in the proliferation of progenitor cells of the basal layer, leading to a thinned epidermis during embryonic development. Moreover, it should be considered that the mTOR pathway is also involved in the epidermal growth factor receptor action, which is a potent activator of many kinases, including serine/threonine kinase mTOR.^38^ In the photoexposed skin specimens of all the study groups, when compared with photoprotected skin, epidermal hyperplasia was observed resulting from the greater proliferation of keratinocytes, which confirms that this type of pathological alteration in the skin is mediated by UVR-B. This alteration was more prominent in photoexposed skin of the MPA and AZA groups. These data related to the greater thickness of the stratum spinosum of the epidermis in an area of photoexposed skin is not an unusual finding, and this reactional effect to sun exposure has already been well described. One study showed that stimulation of the epidermis by UVR-B promotes hyperproliferation of basal layer cells in the cones and in the segments of the epidermis between the cones, with an increase in the number of cell layers.^39^ The higher epidermal proliferation seen in the photoexposed skin of the MPA, AZA, and control groups, when compared to the mTORi group, does not exclude the role of UVR-activated mTOR pathway signaling in cell proliferation and of the pro-survival signaling cascades of epidermal keratinocytes from the skin of these patients.

## Conclusions

The histopathological analyses performed comparatively on the skin of RTRs using the mTORi, MPA and AZA immunosuppression regimens, in comparison with a control group, showed that mTORi was superior in relation to the others, regarding the maintenance of some control mechanisms that may be linked to cutaneous carcinogenesis, since only mTORi presented epidermal proliferation. The most unique morphological alteration of this study was the skin epidermal thinning of RTRs treated with mTORi in both photoexposed and photoprotected skin of these patients.

Based on the data obtained in this study, it can be suggested that the use of mTORi therapy, when compared to the use of MPA and AZA may be recommended as the immunosuppressive regimen in patients who already have skin neoplasias or who are at increased risk for developing skin cancers related to photoexposure, such as NMSCs. However, there are not enough data to define whether it would be better to replace MPA or AZA with mTORi in patients with established skin cancer or whether it would be more advantageous to add the medication to the regimen being used. In the case of AZA, specifically, because it is recognized as carcinogenic, it is possible that it is better to suspend it and replace it by mTORi.

## Financial support

This research received financial support from Fundo de Apoio à Dermatologia do Estado de São Paulo – Sebastião Sampaio (FUNADERSP).

## Authors’ contributions

Maria Victória Quaresma: Main Investigator.

Luiz Sergio Azevedo: Design and planning of the study; supervisor of the nephrological part and review of the manuscript.

Elias David-Neto: Coordinator of the service and review of the manuscript.

Mírian Nacagami Sotto: Design and planning of the study; advisor of the histological part and review of the manuscript.

## Conflicts of interest

None declared.
